# A sensitive gold-nanorods-based nanobiosensor for specific detection of *Campylobacter jejuni* and *Campylobacter coli*

**DOI:** 10.1186/s12951-019-0476-0

**Published:** 2019-03-26

**Authors:** Saeed Shams, Bita Bakhshi, Tahereh Tohidi Moghadam, Mehrdad Behmanesh

**Affiliations:** 1 Department of Bacteriology, Faculty of Medical Sciences, Tarbiat Modares University, Jalal-Ale-Ahmad Ave, Tehran, 14117-13116 Iran; 2 Department of Nanobiotechnology, Faculty of Biological Sciences, Tarbiat Modares University, Tehran, Iran; 3 Department of Genetics, Faculty of Biological Sciences, Tarbiat Modares University, Jalal-Ale-Ahmad Ave, Tehran, 14117-13116 Iran

**Keywords:** *Campylobacter* spp., Gold nanorods, Surface plasmon resonance, Nanobiosensor

## Abstract

**Background:**

Campylobacteriosis is a zoonotic infectious disease that can be mostly undiagnosed or unreported due to fastidious *Campylobacter* species. The aim of this study was to develop a simple, sensitive, and quick assay for the detection of *Campylobacter* spp. and taking advantage of the great sensitivity of gold nanorods (GNRs) to trace changes in the local environment and interparticle distance.

**Methods:**

Characterized GNRs were modified by specific ssDNA probes of *cadF* gene. First, the biosensor was evaluated using recombinant plasmid (pTG19-T/*cadF*) and synthetic single-stranded 95 bp gene, followed by a collection of the extracted DNAs of the stool samples. The sensing strategy was compared by culture, PCR, and real-time PCR.

**Results and discussion:**

Analysis of 283 specimens showed successful detection of *Campylobacter* spp. in 44 cases (16%), which was comparable to culture (7%), PCR (15%), and real-time PCR (18%). In comparison with real-time PCR, the sensitivity of the biosensor was reported 88%, while the specificity test for all assays was the same (100%). However, it was not able to detect *Campylobacter* in 6 positive clinical samples, as compared to real-time PCR. The limit of detection was calculated to be the same for the biosensor and real-time PCR (10^2^ copy number/mL).

**Conclusions:**

Taking high speed and simplicity of this assay into consideration, the plasmonic nanobiosensor could pave the way in designing a new generation of diagnostic kits for detection of *C. jejuni* and *C. coli* species in clinical laboratories.

**Electronic supplementary material:**

The online version of this article (10.1186/s12951-019-0476-0) contains supplementary material, which is available to authorized users.

## Introduction

*Campylobacter* spp. are fastidious Gram-negative bacteria which are known as a common cause of human acute gastroenteritis (campylobacteriosis) worldwide [1]. Among various species, *Campylobacter* (*C.*) *jejuni* is a species with more clinical prevalence (about 90%), followed by *C. coli* (about 10%) [2, 3]. The reservoir of the bacteria is mainly poultry, but it can also be transmitted to humans through consumption of animal products, contaminated water, and untreated milk, [4–6]. Although many cases of campylobacteriosis are undiagnosed the disease incidence has been estimated to affect over 1.3 million cases annually [7].

There are several methods (e.g. culture, molecular, and serological tests) for the detection of campylobacteriosis [8–10].

Stool culture is considered as the gold standard for the detection of the disease but often requires several days to complete and sometimes presents unreliable results because of (i) fastidious conditions of the bacteria, (ii) low sensitivity, (iii) and conversion to viable but nonculturable cells (VBNC) [5, 11, 12]. Molecular techniques are other assays for the identification of the *Campylobacter*s with more specificity and sensitivity than culture [13, 14]. Nevertheless, these methods are sensitive to stool, blood, and urine inhibitors (such as bile, heme, and urea, etc.), expensive, and requiring trained staffs [15, 16]. Serological techniques are other platforms which have been reported for the detection of the *Campylobacters*. Unfortunately, cross-reaction between bacterial antigens have been reported [17, 18]. Hence, it is important to develop a simple, sensitive, and quick assay for the identification of *Campylobacter* species.

In recent years, researchers have been considering to improve new biosensors because of their sensitivity, simplicity, and low cost by different nanostructures, including quantum dots, gold nanoparticles, carbon nanotubes, etc. [19, 20]. Gold nanoparticle-based methods have recently attracted significant attention in biotechnological fields for biosensing applications due to outstanding properties. Amongst them, Gold nanorods with two strong longitudinal surface plasmon resonance (SPR) [21] bands, tunable aspect ratio and extreme sensitivity to trace changes in the dielectric properties of the surrounding have been regarded as promising candidates as diagnostic nanobiosensor to analyze different biomarkers [22–26]. Recently, a few studies have reported the design of GNRs-DNA biosensors for the identification of the microorganisms. For example, Hepatitis B virus, *Chlamydia trachomatis*, and Ochratoxin A were detected by GNRs. [22, 24, 27, 28]. DNA-biosensors are able to form a double-stranded hybrid with their complementary sequences, resulting in the aggregation of the GNRs and change of SPR absorption peak [24]. Although several studies have been conducted on detecting *Campylobacters* by the SPR properties [29, 30], this is the first study based on ssDNA-GNRs biosensing system for the identification of both *C. jejuni* and *C. coli* species using specific *cadF* gene, giving a comparison with bacterial culture, PCR, and real-time PCR assays.

## Methods

### Bioinformatics and design of probes and primers

The *cadF* gene of *Campylobacter* was considered as the target gene in all molecular techniques. Deposited partial and complete sequences of the *cadF* gene from *C. jejuni* and *C. coli* (accession numbers NC_022660.1, CP006702.1, GL405235.1, CP017025.1, KC575115.1, AAFL01000010.1, CP007183.1, CP007179.1, CP004066.1, CP007181.1, AEER01000022.1, HE978252.1, CP017029.1, CP006707.2, and CP006709.2) were downloaded from the NCBI GenBank (http://www.ncbi.nlm.nih.gov/), and multiple alignments of sequences were done using CLC Sequence Viewer 7.6 software (CLC bio, Aarhus, Denmark) to determine conserved regions among members of both species. GeneRunner, AllelID, and CLC software were used for designing the probe and primers of real-time PCR assay and probes of the biosensor (Table 1).

**Table 1:** Primers and probes used in this study

Primer name	Sequence (5′ → 3′)	Target	Size	Reference
PCR	F: TTGAAGGTAATTTAGATATG R: CTAATACCTAAAGTTGAAAC	*C. jejuni/C. coli*	400 bp	[49]
Duplex-PCR	FU: TTGAAGGTAATTTAGATATG R1: TTTATTAACTACTTCTTTTG R2: ATATTTTTCAAGTTCATTAG	*C. coli* *C. jejuni*	461 bp 737 bp	[10, 49]
Real-time PCR	F: AACCCAAATTCTAATTGATC R: GAAGGTAATTTAGATATGGATAA P: FAM-AAATGATAACCAAGTCTAATCCCTGG	*C. jejuni/C. coli*	95 bp	This study
Biosensor	Probe 1: TTATCCATATCTAAATTACC-SH Probe 2: SH-AGTCTAATCCCTGGTGCATA	*C. jejuni/C. coli*	44 bp	This study
Cloning	M13F: AGGGTTTTCCCAGTCACGA M13R: GAGCGGATAACAATTTCACAC	Recombinant plasmid (for sequencing)	193 bp	[50]

### Reagents for nanobiosensor

Chloroauric acid (HAuCl_4_·3H_2_O), cetyltrimethylammonium bromide (CTAB), sodium borohydride (NaBH_4_), sodium acetate, and phosphate buffered saline tablet (PBS; 10 mM, pH 7.4) were procured from Sigma Aldrich. Ascorbic acid, silver nitrate (AgNO_3_), sodium chloride (NaCl), and Dithiothreitol (DTT) were purchased from Merck. Probes, primers, and synthetic single-stranded 95 bp *cadF* gene were synthesized by Eurofins MWG Operon (Ebensburg, Germany) company.

### Preparation and characterization of gold nanorods

GNRs were synthesized using the previously described seed-mediated growth method reported by Tohidi Moghadam and Nikoobakht [31, 32]. Briefly, the seed solution was prepared by mixing CTAB (7.5 mL, 0.095 M) and HAuCl_4_·3H_2_O (250 μL, 0.01 M) followed by mixing with fresh ice-cold NaBH_4_ (600 μL, 0.01 M) and rapid inversion for 2 min to form a yellow–brown solution. Keeping the reaction mixture undisturbed at ambient temperature for 2 h, the growth solution was prepared by addition of CTAB (9.5 mL, 0.095 M), HAuCl_4_·3H_2_O (400 μL, 0.01 M), AgNO3 (60 μL, 0.01 M), and ascorbic acid (64 μL 0.10 M) to a test tube sequentially. Then, 40 μL of the seed particles were added to the mixture to initiate the growth of GNRs.

Then, excess CTAB and unreacted gold ions were removed from the solution by two rounds of centrifugation (13,000 rpm, 7 min). The supernatant was carefully decanted, and the pellet containing GNRs was re-dispersed in a PBS buffer (pH 7.4, 10 mM). Prior to bioconjugation with probes, optical density (OD) of the stock GNRs was adjusted to 1. The SPRs characteristic of the GNRs was evaluated by UV–vis spectrophotometer (Perkin-Elmer, Thermo Scientific, USA) in the wavelength region of 400–900 nm. Size and morphology of the nanostructures were analyzed by Zeiss EM900 transmission electron microscopy at an accelerating voltage of 50 kV (ZEISS, Germany).

### Preparation of ssDNA-GNRs nanobioconjugates (Nanoprobe)

The thiol group (–SH) of each probe was reduced by Dithiothreitol (DTT). Briefly, 10 μL of 1.0 N DTT (in 0.01 M Sodium acetate, pH 5.2) was added to the thiolated probes. The mixture was vortexed and incubated at room temperature (RT) for 15 min. To remove DTT, thiolated-oligonucleotide mixture was washed 3 times with 50 µL ethyl acetate. Each time, the upper layer was discarded after vortexing.

A total of 300, 200, 150, 100, 50, and 25 nM of freshly cleaved probes were then added immediately to 250 μL of GNRs buffered to 0.1 M NaCl and incubated for 30 min at RT. After the bioconjugation process, the GNRs were centrifuged and the concentration of the unbound ss-DNAs were measured by spectrophotometer (Eppendorf, Hamburg, Germany). Using ICP-AAS (Inductively coupled plasma-atomic absorption spectroscopy), the concentration of the GNRs was evaluated. Both results were used for estimating the ratio of GNRs:ssDNA. Immobilization of the ssDNA probe on the GNRs was analyzed by Fourier Transform Infrared Spectrometer (FTIR, Thermo Nicolet-Nexus 870, USA), UV–vis spectra, Dynamic Light Scattering (DLS), and zeta potential (Zetasizer Nano ZS, England, Malvern).

### Preparation of recombinant pTG19-T/*cadF* plasmids as a standard control

To provide a standard control for gold nanobiosensor and real-time PCR analyses, a recombinant pTG19-T/*cadF* plasmid was prepared and used according to a protocol described by Soleimani et al. [33]. Briefly, PCR amplification of the *cadF* gene was performed using primers designed for real-time PCR assay (forward primer: AACCCAAATTCTAATTGATC and reverse primer: GAAGGTAATTTAGATATGGATAA). After purification of PCR products by PCR purification Kit (Bioneer, Daejeon, Korea), a 95 bp fragment of *cadF* gene was ligated into a pTG19-T vector according to manufacturer’s instructions (TA Cloning kit, CinnaClon Co., Iran). Extracted plasmids of the cloned *E. coli* TOP10F´ were evaluated by PCR and sequencing using specific primers (M13F and M13R) described in Table 1.

### Initial evaluation of nanobiosensor efficacy by spiking pTG19-T/*cadF* plasmid and synthetic single-stranded 95 bp *cadF* gene in stool samples

The nanobiosensor was initially evaluated by spiking recombinant pTG19-T/*cadF* plasmid and a synthetic single-stranded 95 bp *cadF* gene in *Campylobacter*-free stools. Accordingly, 250 µL of each ssDNA-GNR Probe 1 and 2 solutions (Probes 1 and 2 conjugated to GNRs in 10 mM PBS buffer) were mixed together at RT. Then, the plasmid and the single-stranded 95 bp gene were extracted from spiked samples and the targets (~ 4 ng) were denatured by heating at 100 °C for 15 min in a thermoblock and immediately chilled in ice for another 5 min to obtain denatured single-stranded DNA. After adding DNA targets to the biosensors, the changes in morphology of the GNRs and absorbance peaks of the biosensor were evaluated using transmission electron microscopy at 50 kV and UV–vis spectrophotometry, respectively.

The resultant SPR spectra from the recombinant plasmid and the single-stranded 95 bp gene were compared with extracted DNA from standard bacteria.

### Collection and culture of human stool specimens

In a 1-year period (2016–2017), 283 children under 5-year of age with intestinal signs and suspected to campylobacteriosis, referring to two pediatric hospitals in Tehran, Iran, were enrolled in the present study. Stool specimens were transferred to the bacteriological laboratory into Carry-Blair transport media (Micro Media, Hungry) and immediately cultured on Brucella agar with 5% sheep blood and modified charcoal–cefoperazone–deoxycholate agar (mCCDA) (Merck, Germany) supplemented with specific antibiotics (ibresco, Iran). Incubation was performed at 42 °C for 48 h under microaerobic conditions. Suspicious colonies were evaluated by Gram staining, standard biochemical testing, and PCR using primers reported by Konkel et al. (Table 1).

### DNA extraction of the stool

DNA extraction was performed from all fecal samples using QIAamp DNA Stool Mini kit (Qiagen GmbH, Hilden, Germany) according to the manufacturer’s instruction. Usually, the kit removes all inhibitors of the stool and a pure DNA can be obtained. This can avoid the interferences in the molecular techniques, especially in the biosensor. The extracted DNAs were stored at − 20 °C until further procedures About 4 ng of each extracted DNAs from the patients were uniformly used in all assays.

### Direct evaluation of stool by PCR

A PCR assay was performed on all extracted DNA from stools using primers designed for real-time PCR assay. The PCR was carried out in a 25-μL reaction mixture containing 4 ng DNA template, 2.5 μL PCR buffer 10×, 200 μM dNTP, 5 mM MgCl_2_, 0.1 μM *cadF* primers, 1 unit of Taq DNA polymerase, and sterile deionized water. The cycling conditions were as follows: 95 °C for 3 min (1 cycle), followed by 32 cycles of denaturation at 94 °C for 30 s, annealing at 43 °C for 30 s, and extension at 72 °C for 30 s in a thermocycler (Eppendorf, Hamburg, Germany) with an additional extension step (5 min, 72 °C). To evaluate whether the designed nanobiosensor and real-time PCR can simultaneously detect both *C. jejuni* and *C. coli* species in stool specimens, an alternative duplex-PCR assay was also done on the specimens as previously described [10]. *C. jejuni* ATCC 29428 and *C. coli* ATCC 43478 strains were used as reference strains in the method.

### Direct evaluation of stool by real-time PCR

Evaluation of DNA by real-time PCR assay was carried out using a StepOne instrument (Applied Biosystems, Foster, CA, USA). The real-time PCR reaction was performed in a final volume of 25 µL using the TaqMan Universal PCR Master Mix (Ampliqon, Denmark), containing 250 nM fluorogenic probe, 900 nM of each primer, and 4 ng of template DNA. Thermal cycling conditions were as follows: 50 °C for 2 min, 95 °C for 10 min, 40 cycles of 95 °C for 15 s, and 60 °C for 1 min. In each round of the PCR, the recombinant plasmid was used as positive control.

### Direct evaluation of stool by nanobiosensor

The biosensor efficacy for the detection of the *C. jejuni* or *C. coli* DNA directly in the stool samples was evaluated. The SPR absorbance changes of the assay were immediately evaluated in the presence of the target gene by UV–vis spectrophotometry. The recombinant plasmid was used as positive control. Visual evaluation was also performed by naked eye.

### Limits of Detection (LODs), specificity, and sensitivity of designed nanobiosensor, real-time PCR, and PCR

The LODs of the gold biosensor, real-time PCR, and PCR assays were evaluated using serial 10-fold dilution of the pTG19-T*/cadF* plasmid stock (e.g., 100 µL of plasmid: 900 µL of DNase-free water). The concentration of the diluted DNAs was determined from 4 to 4 × 10^−10^ ng (~ 10^9^ to 1 copy number/mL). Similar initial amounts of ~ 4 ng of plasmid were spiked in the stool and serial dilutions were prepared. After extraction step, LODs of the biosensor, real-time PCR, and PCR methods were evaluated. In all assays, the above-mentioned calculation was performed with different concentrations of the plasmid. The lowest concentration which can be detected by each technique was defined as LODs. To quantify the LOD of biosensor, a standard curve was generated by plotting the *Tt* (time threshold) values against log copy number, and linear regression was considered using the Microsoft Excel program. Each test was performed in independent triplicates.

A collection of the genomic DNA of enteropathogenic bacterial strains, e.g. *E. coli* ATCC 25922, *Vibrio cholerae* ATCC 14035, *Aeromonas hydrophila* ATCC 7966, *Enterobacter cloacae* PTCC 1798, *Shigella sonnei* ATCC 9290, *Campylobacter jejuni* ATCC 29428*, Campylobacter coli* ATCC 43478, and clinical isolates of Enteropathogenic *E. coli*, Enterohemorrhagic *E. coli*, and *Salmonella enterica* were used for specificity test. The DNAs of the strains were extracted using an EZ-10 Spin Column Genomic DNA Mini-Preps Kit (Bio Basic Inc., Canada) according to the manufacturer’s instruction. Used concentrations of the DNAs for all assays were adjusted to ~ 4 ng and the protocol was done with the above- mentioned identical conditions. The sensitivity and specificity of three assays for direct detection of the *Campylobacters* in fecal specimens was calculated according to presented formula by Parikh et al. [34]. In sensitivity tests, the real-time PCR method was used as gold standard.

### Stability of biosensor

In order to investigate the stability, the GNR-ssDNA biosensor was kept at 4 °C for a period of 6 months and was monitored by UV–vis spectrophotometer. The specificity and the LOD of nanobiosensor were also evaluated during the stability period by the recombinant plasmid and non-target DNAs. The done conditions for the stability assays were similar to the above-mentioned conditions. All experiments on the clinical samples were carried out with fresh nanobiosensor.

## Results and discussion

### TA cloning and positive control

**Fig. 1:**
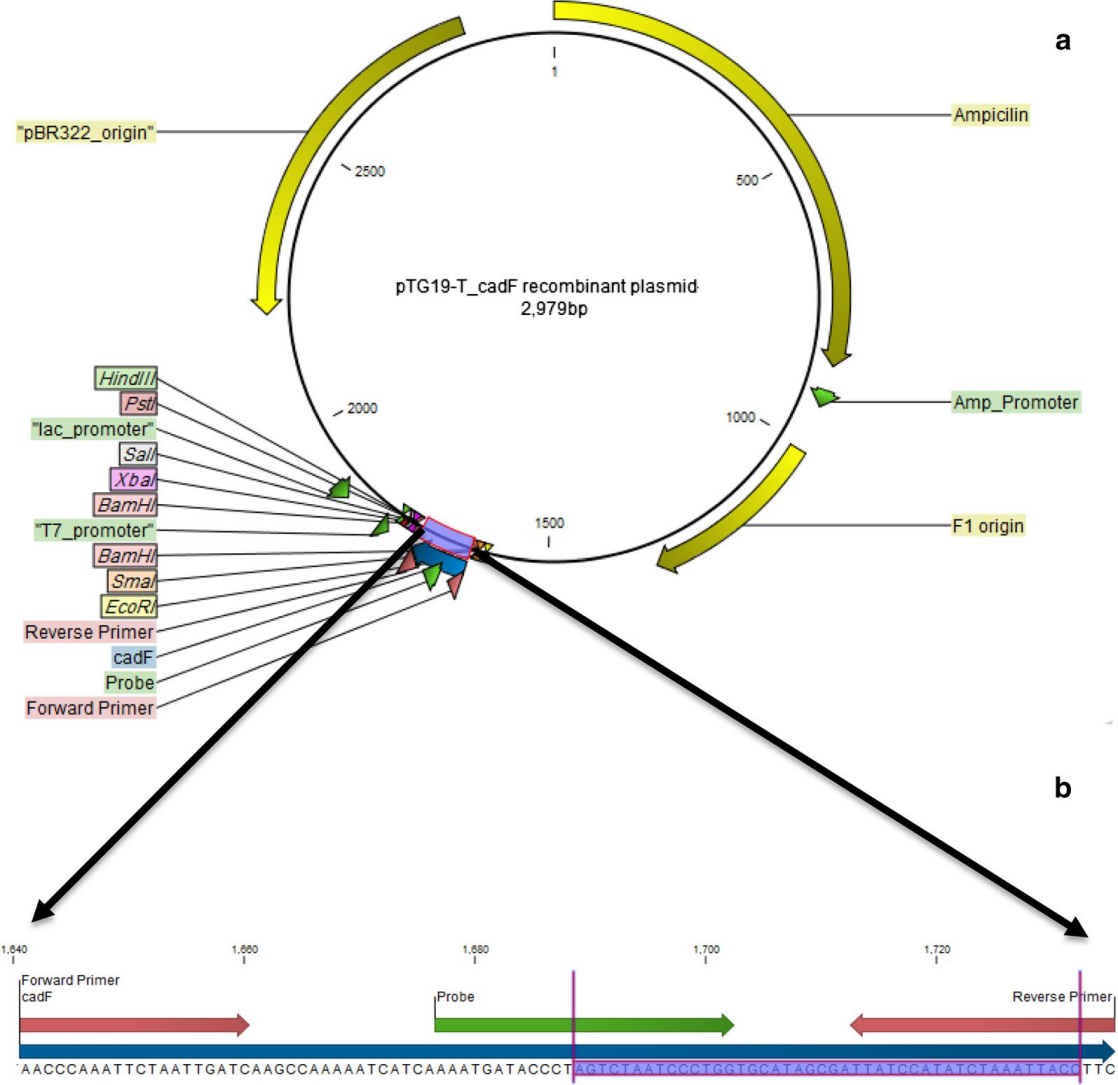
Schematic map of the positive control. A; pTG19-T/*cadF* recombinant plasmid + 95 bp fragment of *cadF* gene inserted into vector (blue box). B: The attachment position of probe/primers used in real-time PCR (red and green arrows) and target sequence of biosensor (blue scheme)

TA cloning of *cadF* gene following recombinant plasmids sequencing confirmed that target fragment was correctly assembled into the vector and can efficiently hybridize with target gene from both *Campylobacters*. The position of the cloned *cadF* fragment and biosensor target is presented in Fig. 1.

### Synthesis and characterization of nanorods

**Fig. 2:**
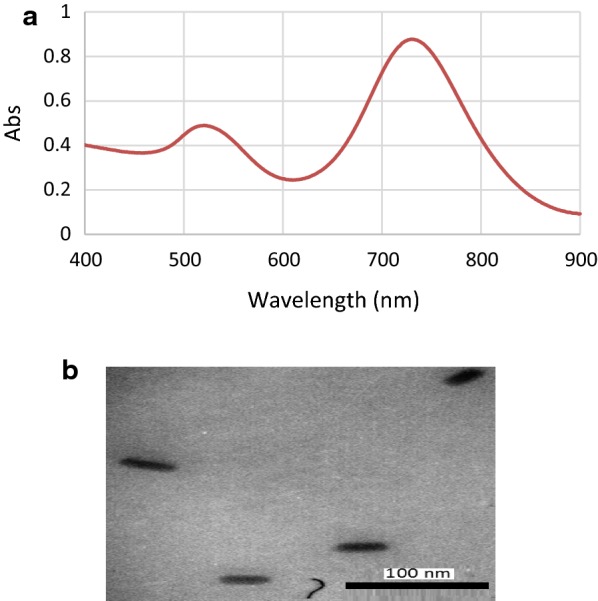
Synthetized nanorods. **a** Characteristic surface plasmon resonance bands of GNRs; **b** the inset shows TEM of the nanostructures

Compared with spherical gold nanoparticles, gold nanorods show greater absorption cross sections and stronger light scattering properties, giving two absorption peaks in the visible and near-infrared (NIR) regions that correspond to the transverse and longitudinal surface plasmon bands, respectively. In this study, the characteristic transverse absorption band appeared at ~ 500 nm while the longitudinal absorption band emerged around 730 nm, representing the formation of rod-shaped nanostructures. Analysis of transmission microscopy also confirmed the rod morphology and size of the GNRs with the average length and the diameter of ~ 32.43 ± 5.5 nm and ~ 11.53 ± 1.03 nm, respectively. Size of the nanostructures was estimated by ImageJ Software (Fig. 2b).

### Analysis and optimization of GNRs-ssDNA nano-bioconjugates

Surface functionalization of gold with ssDNA has been reported previously [35]. Thiol activated ssDNA is usually utilized for adsorption onto the gold surface with high affinity. Formation of the ssDNA-GNRs complex was first monitored by UV–vis spectroscopy. Although it is very important to maximize DNA loading onto GNRs, due to the high sensitivity of these nanostructures care must be taken to maintain the rod morphology during the bioconjugation process. Upon the bioconjugation process with different concentrations of the probe (25–300 nM), the characteristic SPR bands of GNRs experienced a decrease in intensity. The results showed that addition of higher concentrations of the probe (above 25 nM) has led to the disappearance of typical SPR bands, representing a nonspecific aggregation of the nanostructures. Therefore, the specified concentration was used for further experiments to have a proper control on interparticle distance before target addition without losing the characteristic morphology as well as showing stability over a period of time. Such an optimization is of great significance in the process of design and development of SPR based nanobiosensors [32]. The GNRs were centrifuged after the bioconjugation process (with 25 nM DNA) and concentration of the unbound ss-DNAs was measured to be ~ 8 nM. Using ICP-AAS, the concentration of the GNRs can also be calculated. Based on the average length and diameter of the GNRs (to obtain the number of atoms in each GNR) and ICP results, molarity of GNRs in this study was calculated to be 1200 pM. Therefore, the ratio of GNRs:ss-DNAs in which the nanostructures can maintain their rod morphology was calculated to be 1.2: 17.

**Fig. 3:**
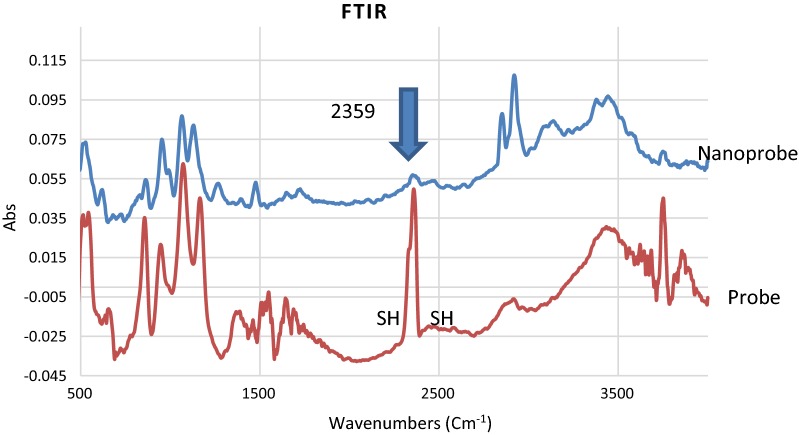
FTIR spectra of the nanoprobe before and after immobilization onto GNRs

Figure 3 shows that the absorption peak of the FTIR around 2359 cm^−1^ can be correlated to –SH group of the probes. Upon immobilization of the probes onto the matrix of GNRs, the peak disappeared, resulting the attachment of –SH to Au [36]. It is worth mentioning that the strong absorption bands around 2920 and 2850 cm^−1^ in the nanoprobe sample appeared due to C–H stretching vibrations of methyl and methylene groups of CTAB (the cationic surfactant) on the matrix of GNRs. The small band around 1470 cm^−1^ can also be attributed to C-H bending vibrations of the CTAB layer around GNRs [37].

Moreover, to monitor the interaction of the probes with GNRs, zeta potential of the samples before and after the bioconjugation process was analyzed. The zeta value for the GNRs decreased from + 30.1 to + 15.8 mV after interaction with ssDNA, indicating changes on the surface of GNRs (Additional file 1). Polydispersity index (PdI) for both nanostructures were ~ 0.4 (Additional file 2).

### Analysis of nanobiosensor in the presence of recombinant plasmid and synthetic 95 bp *cadF* gene

**Fig. 4:**
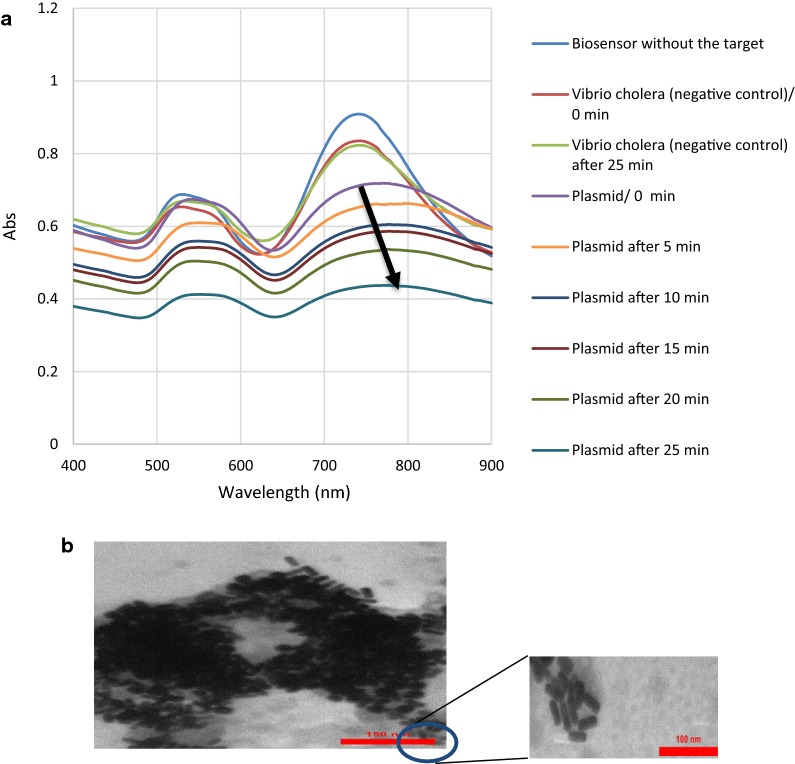
Nanobiosensor upon addition of recombinant plasmid as the target gene. **a** surface plasmon resonance bands; **b** transmission electron microscopy image

Figure 4 shows the SPR bands and TEM image of the nanobiosensor after hybridization with recombinant plasmid of *cadF* gene of *Campylobacter*. Upon addition of the extracted genomic DNA, the shape of the SPR bands of the nanoprobes apparently changed. This phenomenon is thought to have occurred due to the hybridization of *cadF* specific sequence with capture probes that are complementary to each other, leading to the notable decrease of interparticle distance. A glance at the SPR response of the nanobiosensor shows the significant decrease in the intensity as well as red-shifts in the position of the longitudinal surface plasmon absorption bands upon aging (up to 25 min) and final loss of the SPR bands. This could be attributed to the change in the refractive index at the surface layer of the GNRs, induced by binding to the target [38]. The very first change can be observed after immediate addition of the target DNA to the nanoprobe. A decrease in the intensity of the transverse plasmon band was also detected around 500 nm. The SPR bands of the *Vibrio cholerae* DNA, as the negative control, showed no changes upon interaction. Transmission electron microscopy image (Fig. 4b) revealed the remarkable decrease of interparticle distance and aggregation of the nanoprobe after hybridization with the target.

**Fig. 5:**
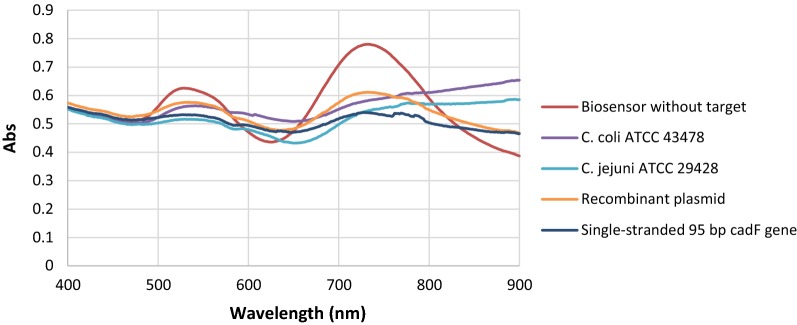
SPRs spectra of the DNAs from standard strains, the recombinant plasmid, and the single-stranded 95 bp *cadF* gene

The same procedure was performed to assess the efficacy of the nanobiosensor against the stool samples spiked with synthetic single-stranded 95 bp *cadF* gene. Upon addition of synthetic single-stranded 95 bp *cadF* target, the SPR response of the nanobiosensor was changed immediately; however, the reaction became stronger within 25 min. Accordingly, the results were similar regarding recombinant plasmid as the target and indicated the biosensor efficacy for the detection of *cadF* gene of *Campylobacter* in stool samples. In addition, SPRs spectra were compared for the three positive controls including the DNAs from standard strains, the recombinant plasmid, and the single-stranded 95 bp *cadF* gene. Our results showed that plasmonic changes were identical for three targets (Fig. 5). Therefore, it seems that the current biosensor can be directly applied for the detection of the *Campylobacter* in the fecal specimen of patients suspected to campylobacteriosis.

### Comparison of LOD, sensitivity, and specificity of the nanobiosensor with PCR and real-time PCR and evaluation of stability tests

Ten-fold dilutions of pTG19-T/*cadF* recombinant plasmid were used to determine the detection limit of the molecular methods. According to the complete sequence of cloned plasmid (2884 + 95 = 2979 bp) and copy number formula, LODs of PCR, real-time PCR, and biosensor were calculated. Although the biosensor was stable when the recombinant plasmid concentrations were 40 × 10^−9^ and 40 × 10^−10^ ng, the aggregation of gold nanoprobes took place after adding 40 × 10^−1^ to 40 × 10^−8^ ng of DNA. Consequently, the 10^−8^ dilution (10^2^ copy number/mL) was determined as LOD of the biosensor. The LOD values for biosensors are different in published studies. Singh et al. reported 50 copy number. mL^−1^ for the detection of *E. coli* O157:H7 by gold nanorods while Wei et al. described 10^3^ copy number. mL^−1^ of *Campylobacter* using a SPR biosensor [30, 39].

**Fig. 6:**
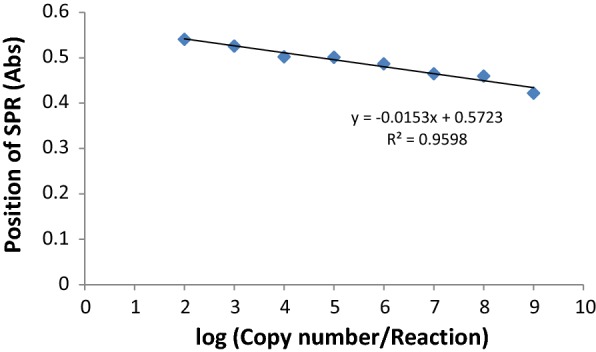
Standard linear regression curve of log (copy number/reaction) against SPR position

Figure 6 indicates the standard linear regression curve of log copy number/reaction against SPR position with a significant difference in the intensity of the SPR between 10^9^ and 10^2^ copy number of target DNA. The changes in the SPR position was linearly observed when the plasmid increased with a correlation coefficient of ~ 0.95. Considering the concentration of *C. jejuni* during human infection which can reach as high as 10^8^ copy number/g in fecal material (3), the biosensor can efficiently detect the target with a remarkable change in the SPR absorption peak. In this study, the broadening of the longitudinal surface plasmon resonance band and disappearance of characteristic LSPR band can be correlated to the presence of the target (Fig. 6).

The detection limit of the PCR and real-time PCR was also detected as 10^−7^ and 10^−8^ dilutions; equal to ~ 10^3^ and ~ 10^2^ copy number/mL, respectively. It was found that LODs of the nanobiosensor and real-time PCR were the same, and it seems that this system can be applied as a potential tool in the genomic hybridization assay for diagnostic purposes. Considering the technical basis of the mentioned methods which are DNA targeted, analysis of their higher sensitivity should be interpreted with cautious as DNA targeted diagnostic tools cannot discriminate between viable and dead cells. Although, considering the pathogenic nature of *C. jejuni* and *C. coli* which are not categorized within normal flora population, makes its DNA detection confirmative of the disease. The detection limit of the methods on spiked stool was 10^−6^, 10^−7^ and 10^−7^ for PCR, real-time PCR, and nanobiosensor, respectively, each of which is one-fold higher than what was obtained for spiked DNase-free water. The highest sensitivity for the detection of *Campylobacter* spp. in clinical samples was related to real-time PCR (50 of 50, 100%). This technique was used as the standard to assess the sensitivity of other assays. Higher sensitivity (100%) of real-time PCR was also reported in Zhang study [40]. When compared with real-time PCR, the sensitivity of the biosensor and PCR were reported as 88% (44 of 50) and 84% (42 of 50), respectively. Our nanobiosensor showed an acceptable sensitivity range between PCR and real-time PCR which proposes its application for development of a promising diagnostic tool in the upcoming future.

**Fig. 7:**
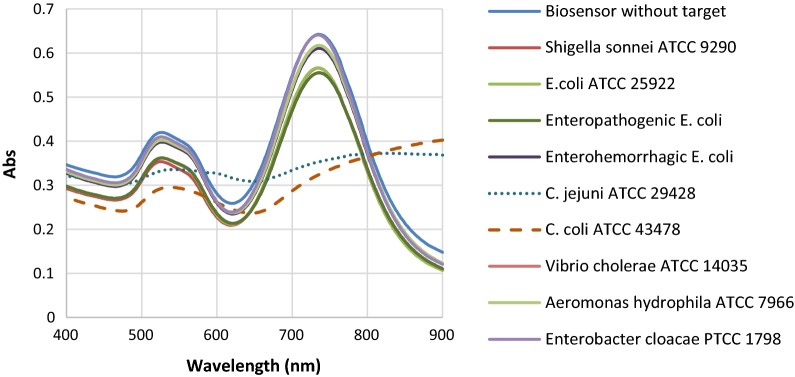
Specificity results of the nanobiosensor

For specificity evaluation, a list of intestinal bacteria was used for PCR, real-time PCR, and biosensor. The specificity results for all 3 tests were 100% with exclusive detection of both *C. jejuni* and *C. coli* species and no non-*Campylobacter* detection in the 3 test (data not shown). Changes in the SPR absorption peak of the nanobiosensor was also specific for standard strains of *Campylobacter* while no remarkable variation was found for the other non-*Campylobacter* enteric bacteria (Fig. 7).

**Fig. 8:**
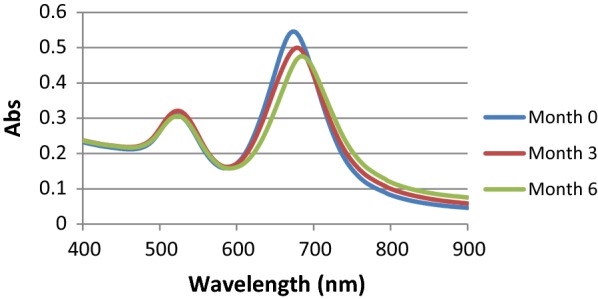
Stability of the gold nanostructure under a period of time

Based on stability findings, there was no aggregation of the nanostructures, and the sensor could maintain its characteristic rod morphology. A small shift in the longitudinal surface plasmon resonance of the GNRs represents some changes in their microenvironment which is mainly size (Fig. 8). Therefore, the nanbiosensor reported in this study was stable over this period of time.

During stability time, LOD of the nanobiosensor were also evaluated by recombinant plasmid and was calculated as ~ 10^2^ copy number/mL which was similar to freshly prepared nanobiosensor. In the specificity test on the control biosensors, no measurable change of the SPR was observed when non-target DNAs were used. The aggregation was only identified in the samples with recombinant plasmid and synthetic single-stranded 95 bp gene. All these data supported that the designed sensor is highly stable, specific, reliable, and sensitive for the detection of *Campylobacter*.

### Culture of clinical specimens

According to morphological and phenotypical characterizations, total numbers of *Campylobacter* spp. positive clinical specimens were 19 out of 283 cases (7%), including 17 *C. jejuni* (89%) and 2 *C. coli* species (11%). Ghorbanalizadgan et al. [41] isolated *Campylobacter* spp. in 12 out of 200 evaluated children (6%) in Iran. Other studies also published data on the isolation rate of *Campylobacter* spp. between 0 to 16% from clinical stool sample by culture in Iran and some other countries [9, 42].

### Detection of *Campylobacter* spp. in stool by direct PCR

Using direct PCR, *cadF* gene was detected in 42 (15%) patients. This technique identified bacterial DNA in 19 out of 19 (100% sensitivity) culture-positive specimens and in 23 culture-negative samples. In bacteriology, the culture is the gold standard of the detection of the bacteria, but some those such as *Campylobacters* aren’t able to grow properly on the media because of fastidious conditions, VBNC, etc. Therefore, despite the disease, the bacteria cannot be isolated in culture method. However, the culture only can detect the live bacteria, while molecular methods can detect DNA from both dead and live bacteria. So it is normal that higher rates of the positive cases can be detected by the molecular tests [43].

O’Leary et al. [44] reported that *Campylobacter* detection by PCR is more sensitive than culture. Singh et al. [45] also indicated that PCR could detect *C. jejuni* more efficiently than culture method.

In addition, all specimens were examined to test the presence of target gene using duplex-PCR described in our previous study [10], which could simultaneously detect and differentiate *C. jejuni* and *C. coli* species. Its result was similar to the common PCR (42 positive cases, including 40 *C. jejuni*, 95%, and 2 *C. coli* species, 5%).

### Evaluation of campylobacteriosis in fecal samples by direct real-time PCR

In this study, a real-time PCR was developed using new TaqMan probe and primer sets, which were able to amplify a 95 bp fragment of the *cadF* gene with accurate specificity confirmed by direct sequencing of the real-time PCR products and analysis of results in NCBI, PubMed (http://www.ncbi.nlm.nih.gov/). With real-time PCR, 50 (18%) out of 283 clinical samples were positive for *Campylobacter* spp. The assay was also able to identify 19 of 19 and 42 of 42 culture and PCR-positive specimens, respectively. Moreover, the designed real-time PCR showed 100% sensitivity compared with two other methods. However, another potential benefit of real-time PCR test over the standard PCR and culture was a short time (about 40 min) needed for the detection whilst the minimum times for PCR and culture, with confirmatory tests to identify a *Campylobacter* isolate at the species level, were about 3 h and 5 days, respectively. This finding is in agreement with De Boer et al. [47] and Zhang et al. [46] studies, which showed the sensitivity of real-time PCR for the identification of *Campylobacters* was both higher and faster than culture method [40, 47].

### Direct detection of *Campylobacter* spp. in stool samples by the nanobiosensor

**Fig. 9:**
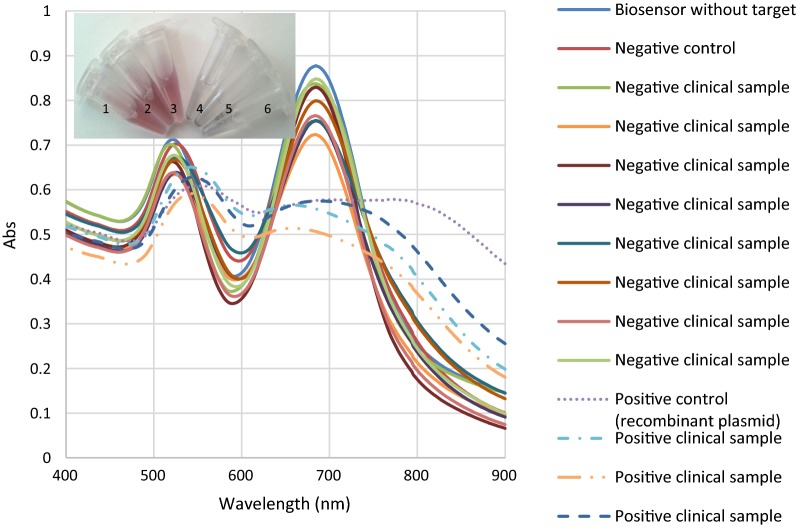
SPR bands of the nanobiosensor upon interaction with different clinical enteropathogenic bacterial strains. The inset shows naked eye detection of *Campylobacter* DNA. 1: negative control, 2 and 3: negative clinical samples, 4 and 5: positive clinical samples, 6: positive control

Of 283 evaluated specimens by the biosensor, *C. jejuni* and *C. coli* species were detected in 44 cases (16%). This method showed higher sensitivity (100%) than PCR (95%) and culture (43%), when it was considered as gold standard. In comparison with real-time PCR, our nanobiosensor was not able to detect *Campylobacter* in 6 positive clinical samples which were also negative in culture method. As shown in Fig. 9, no change in curve shapes of the SPR was observed in the negative clinical specimens, while significant changes representing a specific aggregation of the nanoprobes were observed in the presence of both the target *Campylobacter* DNA and recombinant plasmid. Overall, in DNA genosensing system, there was 12% false negative. The system colour changes were readily observable in the presence of target DNA even by the naked eye (the inset of Fig. 9). This result is consistent with the report by Su et al. [48], developing a colorimetric biosensor for the detection of *E. coli* O157:H7.

## Conclusion

In summary, the present study focused on the design and development of the biosensor for the detection of both target species in human. Similar to real-time PCR, as a standard method, the detection level of the biosensor was 10^2^ copy number/mL, being lower than that of the PCR assay. The false negatives of the nanobiosensor were an unexpected problem, but the system with an acceptable sensitivity and specificity could be useful for the identification of the *Campylobacter* spp. in the clinical setting due to its simplicity, high speed, cost-effectiveness, and portability. It also seems that these advantages can convert the biosensor to a commercial production in the future.

## Additional files

**Additional file 1.** Zeta potential analysis of nanostructures: **A)** GNRs before conjugation with probe; **B)** ssDNA-GNRs nanobioconjugates (nanoprobe).

**Additional file 2.** DLS analysis of nanostructures. **A)** bare GNRs; **B)** nanoprobes.

## Abbreviations

GNRs: gold nanorods
*C*: *Campylobacter*
VBNC: viable but nonculturable cells
SPR: surface plasmon resonance
PCR: polymerase chain reaction
CTAB: cetyltrimethylammonium bromide
NaBH_4_: sodium borohydride
PBS: phosphate buffered saline
AgNO_3_: silver nitrate
NaCl: sodium chloride
DTT: dithiothreitol
UV–vis: ultraviolet–visible
ss-DNA: single-stranded DNA
FTIR: Fourier transform infrared spectrometer
LOD: limit of detection
ATCC: American type culture collection
PTCC: Persian type culture collection
NIR: near-infrared
mV: millivolt
DNA: deoxyribonucleic acid
TEM: transmission electron microscopy
