# Supplementary material for: A sensitive gold-nanorods-based nanobiosensor for specific detection of Campylobacter jejuni and Campylobacter coli
Source: J Nanobiotechnology. 2019 Mar 26;17:43. doi: 10.1186/s12951-019-0476-0 (PMC6434641; doi:10.1186/s12951-019-0476-0)

**Additional file 1.**

Zeta potential analysis of nanostructures: **A)** GNRs before conjugation with probe; **B)** ssDNA-GNRs nanobioconjugates (nanoprobe)

**A)**


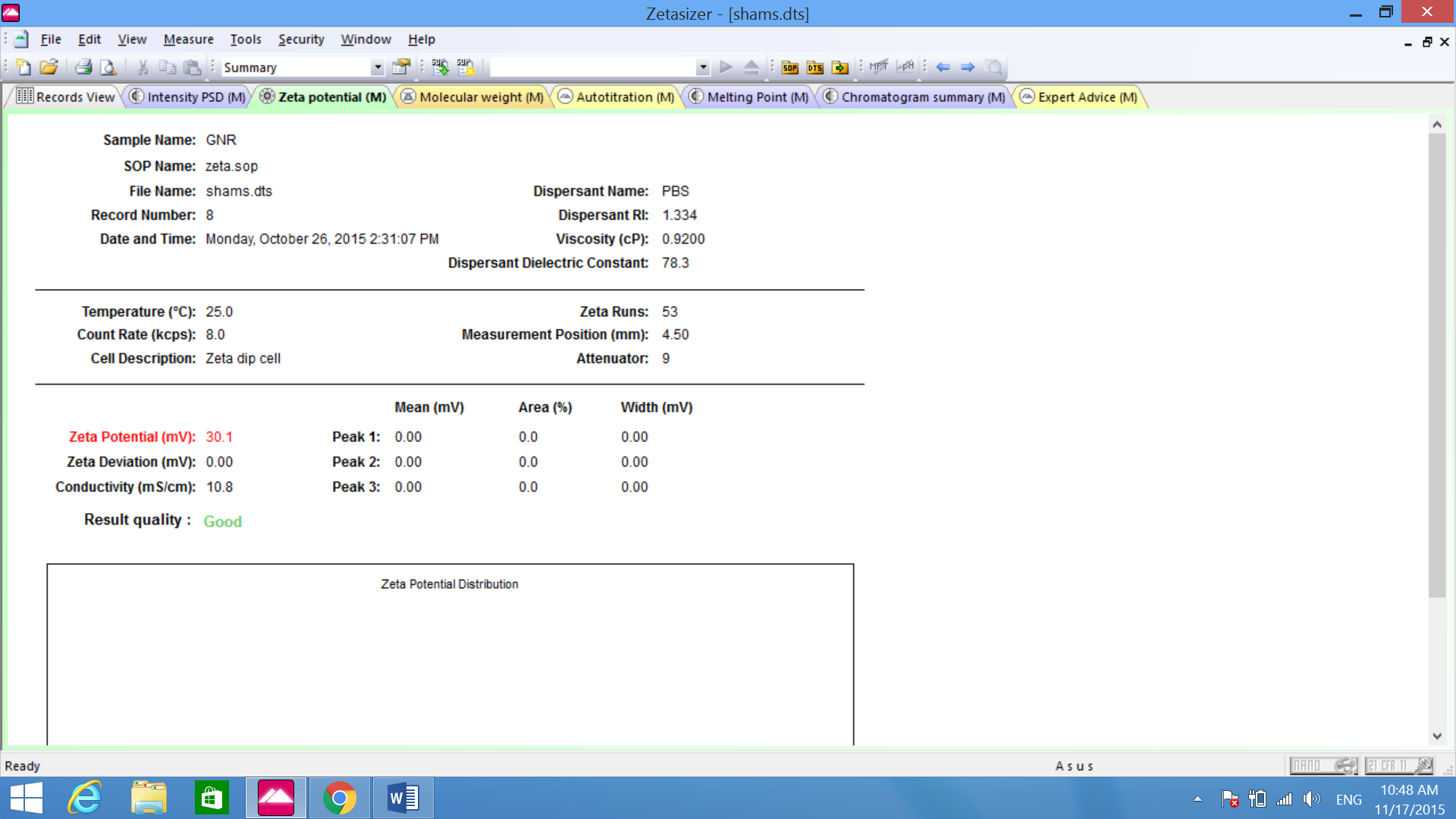


**B)**


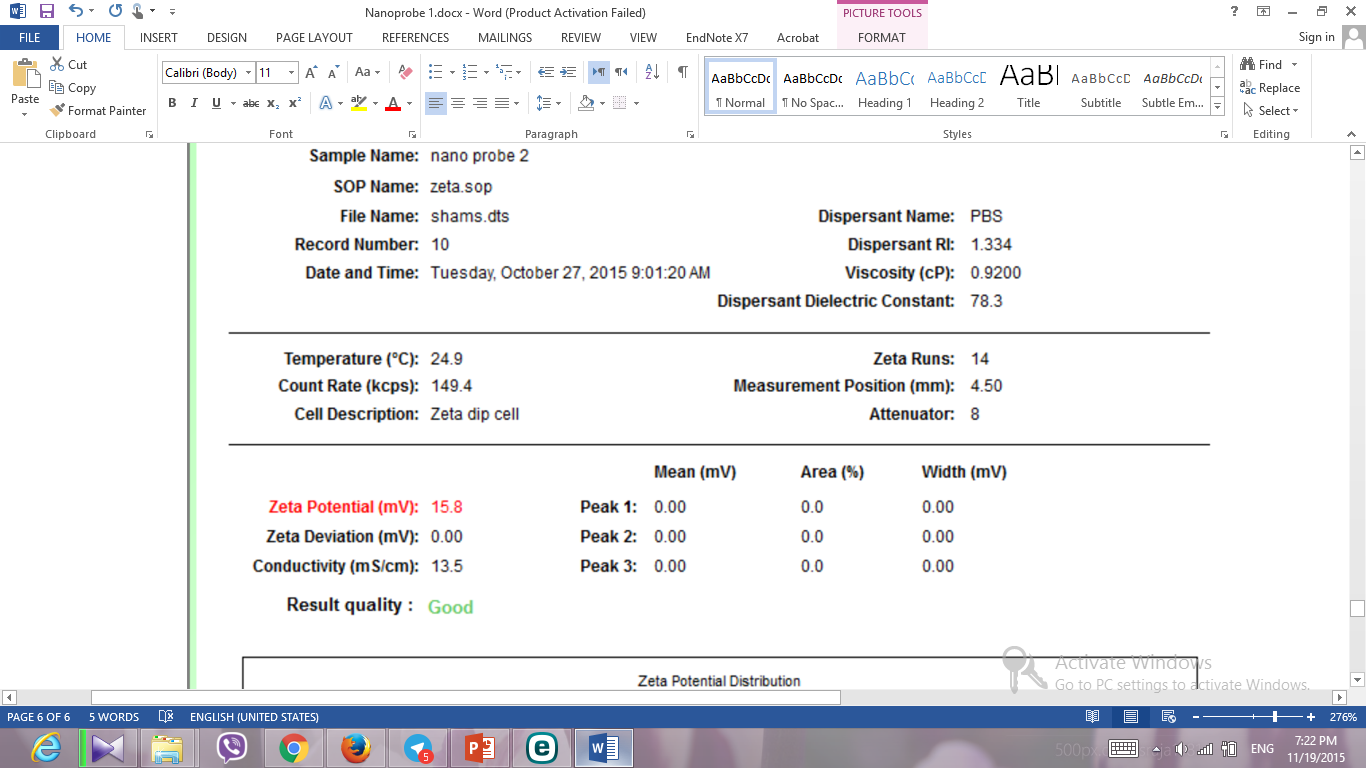

Supplement: Supplementary file 1 — Additional file 1. Zeta potential analysis of nanostructures: A) GNRs before conjugation with probe; B) ssDNA-GNRs nanobioconjugates (nanoprobe). [file 12951_2019_476_MOESM1_ESM.docx]
