# Supplementary figures and images for: A sensitive gold-nanorods-based nanobiosensor for specific detection of Campylobacter jejuni and Campylobacter coli
Source: J Nanobiotechnology. 2019 Mar 26;17:43. doi: 10.1186/s12951-019-0476-0 (PMC6434641; doi:10.1186/s12951-019-0476-0)

**Additional file 2.**

DLS analysis of nanostructures. **A)** bare GNRs; **B)** nanoprobes

**A)**


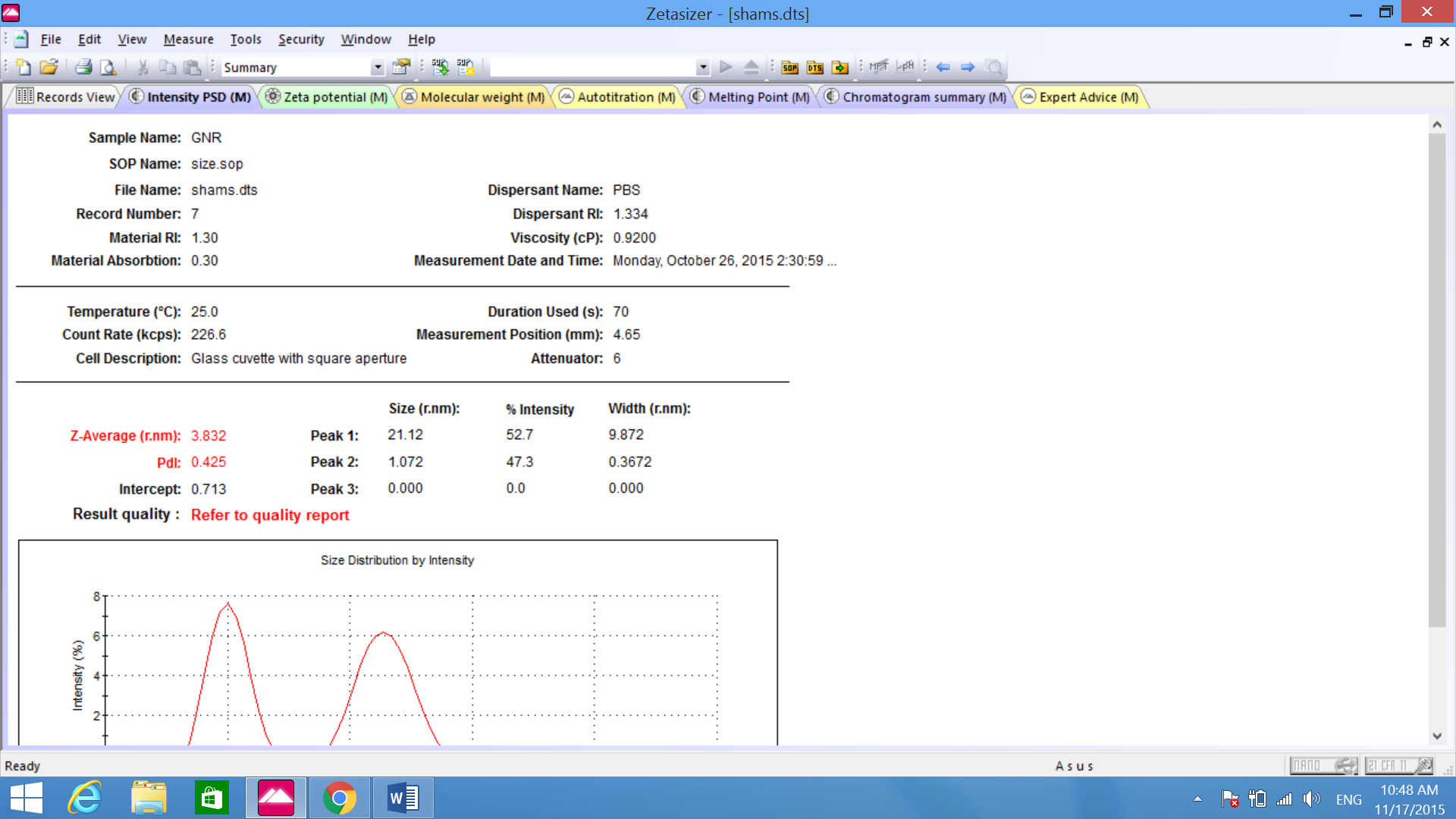


**B)**


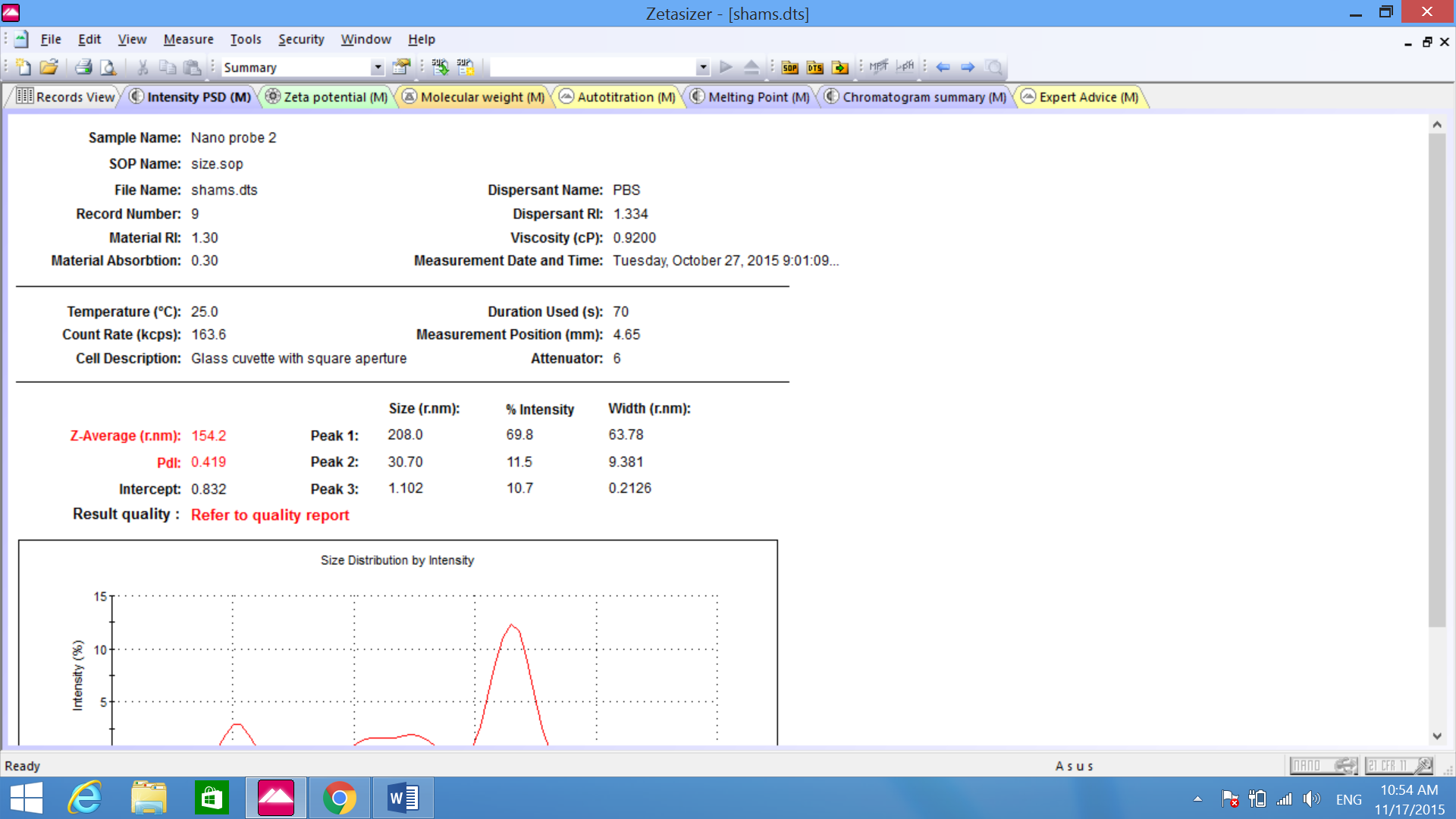

Supplement: Supplementary file 2 — Additional file 2. DLS analysis of nanostructures. A) bare GNRs; B) nanoprobes. [file 12951_2019_476_MOESM2_ESM.docx]
